# Assessing the Effect of Slope Position on the Community Assemblage of Soil Diazotrophs and Root Arbuscular Mycorrhizal Fungi

**DOI:** 10.3390/jof9040394

**Published:** 2023-03-23

**Authors:** Dan Xiao, Tao Hong, Meifeng Chen, Xunyang He, Kelin Wang

**Affiliations:** 1 Pingguo Guangxi, Karst Ecosystem, National Observation and Research Station, Pingguo 531400, China; 2 Key Laboratory of Karst Ecosystem and Treatment of Rocky Desertification, Ministry of Natural Resources, Institute of Karst Geology, CAGS, Guilin 541004, China; 3 Key Laboratory of Agro-Ecological Processes in Subtropical Region, Institute of Subtropical Agriculture, Chinese Academy of Sciences, Changsha 410125, China; 4 Huanjiang Observation and Research Station for Karst Ecosystems, Chinese Academy of Sciences, Huanjiang 547100, China; 5 Guangxi Industrial Technology Research Institute for Karst Rocky Desertification Control, Nanning 530001, China; 6 Guangxi Key Laboratory of Karst Ecological Processes and Services, Huanjiang 547100, China

**Keywords:** diazotroph, arbuscular mycorrhizal fungi, shrub, slope position, soil and root, karst ecosystems

## Abstract

Considering the crucial role of soil diazotrophs and root arbuscular mycorrhizal fungi (AMF) in soil nutrient cycling during ecosystem restoration, diazotroph and AMF communities may be determined by slope position. However, the effect of slope position on diazotroph and AMF abundance, diversity, and community composition of karst ecosystems remains unknown. In this study, soil diazotrophs and root AMF characteristics on varying slope positions were assessed in a karst shrub ecosystem. The results displayed that the abundance of soil diazotrophs and root AMF diversity were significantly affected by slope position. Diazotroph abundance accompanied by soil nutrient and plant richness was higher on the lower slopes than the upper slopes, whereas root AMF diversity displayed the opposite trend. The soil diazotroph and root AMF community composition differed among the upper, middle, and lower slopes. The dominant taxa of soil diazotrophs and root AMF at the order level were Rhizobiales and Glomerales, respectively. Moreover, the diazotroph order of Nostocales and the AMF order of Paraglomerales were richer on the upper slopes than on the lower slopes. The slope position directly affected the plant diversity and soil nutrient distribution, indirectly affecting the diazotroph and AMF communities. Increased available nitrogen on the lower slope caused great diazotroph abundance by stimulating plant growth with sufficient carbohydrates. However, low soil nutrients and plant diversity but high plant root biomass induced more root AMF diversity on the upper slope than on the lower slope. Therefore, this study expands the knowledge of soil diazotroph and root AMF ecological functions along different slope positions during vegetation recovery for the successive stages of grass and shrub in the karst region.

## 1. Introduction

Diazotrophs and arbuscular mycorrhizal fungi (AMF) are important nutrient cycling regulators, particularly during nitrogen (N) progression [1,2,3]. Free-living N fixation can provide N input when the ecosystems lack symbiotic N fixation [2]. The AMF can be symbiotic with most plant roots (80%), and the AMF community in plant roots plays a key role to provide benefits to their host plant by enhancing nutrient use efficiently [4]. The AMF serve as an important bridge between soil and plants, and N fixed from soil diazotrophs can be transferred to plants via AMF [5,6]. The complex environment of the rhizosphere, with rich root exudates, results in strong microbial activity, which strengthens AMF colonization of roots [7,8]. Consequently, AMF species prevail inside the plant root cortex compared with that in the soil. Current studies mainly focus on the evaluation of diazotrophs and AMF in the soil [6,9]. However, less attention has been paid to the study of AMF species in plant roots, and the root diversity and community composition remains unclear. A series factor (e.g., nutrient availability and plant diversity) performs an important role in driving changes in abundance, community composition, and diversity of soil diazotrophs and root AMF. Nevertheless, few studies have focused on the key factor that affects soil diazotrophs and root AMF communities under different slope positions [2,3,4,5,6].

Slope position governs the plant community, nutrient content, soil moisture, and light, which may be closely linked with the richness and composition of diazotrophs and AMF [10,11,12]. For example, a study found that higher AMF diversity occurred on the upper slope compared to the lower slope because sufficient light promoted more AMF species on the upper slope [12]. Many studies have found high nutrient (e.g., soil organic carbon (SOC) and total N (TN)) enrichment on the lower slope with erosion and deposition [13,14]. In contrast, SOC may decrease from slope to valleys in some humid tropical areas [15]. Different soil nutrient response to slope position may be due to variations in litter quantity and quality [15]. The N fixation activity would be suppressed under sufficient N availability conditions [2]. Thus, high N on the lower slope decreases N fixation activity, which reduces the dependence of diazotrophs on AMF. However, low N fixation activity does not necessarily decrease diazotroph abundance. A high nutrient content increases plant diversity. Soil rich in carbohydrates during plant growth provides energy for microbes, thereby improving diazotroph and AMF growth with a high population [16,17,18]. The plant diversity and nutrient availability distribution differed across slope positions, affecting AMF in plant roots and soil diazotrophs [12,19]. However, the effects of plants and nutrients on diazotrophs and AMF, and the extent that slope position affects diazotroph, AMF diversity, and community composition in fragile ecosystems is still unclear.

Karst ecosystems are widely distributed in southwest China [20]. The soil layers in this area are shallow and discontinuous. In such cases, the distribution of soil nutrients and vegetation communities are diverse, especially at different slope positions [21,22]. Strong human disturbances have severely damaged the vegetation in the karst region. Several ecological restoration projects, such as “Grain for Green”, have been implemented to restore degraded ecosystems [21]. The seriously degraded croplands across slopes have been abandoned and restored to shrubs in recent decades [22]. Shrub ecosystems are widely distribution in southwest China, occupying approximately 6.6% of the total area (based on unpublish data). Thus, identifying the contribution of functional microorganisms for diazotrophs and AMF in karst shrub restoration can help promote vegetation succession. Many studies have focused on the characteristics of soil diazotrophs and AMF in karst ecosystems [23,24,25,26]. For example, previous studies found that diazotrophs and AMF in karst soil are dominated by *Bradyrhizobium* and *Glomus*, respectively [23,24,25]. The interaction between diazotrophs and AMF taxa (e.g., *Bradyrhizobium* and *Glomus*) was stronger in karst forests than in non-karst forests [26]. However, diazotroph taxa in soil and AMF groups in plant roots are uncertain in karst ecosystems with different slope positions because of their high habitat heterogeneity, which is followed by changes in soil nutrients and plant diversity.

The aim of the study was to explore the distribution characteristics of soil diazotrophs and root AMF within the upper, middle, and lower slopes to assist in selecting the optimal methods for vegetation recovery. The soil diazotroph abundance, diversity, and community compositions were investigated. In addition, root AMF diversity and community composition were investigated under different slope positions.

## 2. Materials and Methods

### 2.1. Study Site Description

Soil and root samples were collected from nine sites of shrub ecosystems from a typical karst catchment at the Chinese Academy of Sciences: Huanjiang Observation and Research Station for Karst Ecosystems (108°18′–108°19′ E, 24°43′–24°44′ N) in the Guangxi Zhuang Autonomous Region, China. The study area for the karst peak-cluster depression is surrounded by mountains with a steep slope of approximately 30°. The calcareous soil (according to the Food and Agriculture Organization (FAO) soil classification system) in the study region was developed from a dolomite base. The site is characterized by a typical subtropical monsoon climate with an average annual temperature and precipitation of 13 °C and 800–1500 mm, respectively. The rainy season is mainly from May to August, while the dry season is mainly from November to April. This region was naturally restored from cultivated land to a shrub ecosystem over 25 years. The dominant shrubs were *Pyracantha fortuneana*, *Vitex negundo*, and *Alchornea trewioides*. Additionally, other shrubs, such as *Tirpitzia ovoidea*, *Celtis biondii*, and *Mallotus barbatus* were distributed in the study area. Grass, such as *Pteridium aquilinum*, *Euonymus alatus*, and *Pogonatherum crinitum* were also distributed along slope positions.

### 2.2. Experimental Design and Sampling

Three transects were selected at the experiment site, and each transect included three slope position with lower, middle, and upper slopes. Therefore, nine plots (10 m × 10 m) were included in this study (three slope positions × three transects). Plants grow well in the growing season; plant investigation and root samples were determined in July. To ensure comparability, soil samples were also collected in July. Each plot was divided into 25 grids of 2 m × 2 m, and a shrub diversity assessment was carried out in each plot. We also investigated grass community characteristics. Soil samples were collected using a soil drill at the vertex of each grid, and a total of 36 points at a depth of 0–20 cm were thoroughly mixed. Visible stones and residue were removed using a 2.0 mm mesh, and plant roots were collected. The fine roots (<2 mm) were kept at 4 °C within 24 h until rinsed two to three times with distilled water to remove the soil from the surface. Approximately 10 g of rinsed fine roots were placed in a 15 mL centrifuge tube and stored at −80 °C for the microbial index measurement. A portion of the soil sample was stored at 4 °C to analyze soil physicochemical properties, and the remaining soil was stored at −80 °C for DNA extraction. The samples were kept at 4 °C within 5 h prior to the −80 °C storage. AMF is crucial for the acquisition of nutrients, especially in the plant root due to the symbiotic relationship between the plant and AMF. The genetic diversity of coexisting rhizobia was low based on the plate culture and monoclones [27]; thus, we only examined free-living diazotroph diversity and community composition from the soil, rather than rhizobia from root nodules. Overall, in the current study, AMF sequencing was assessed in plant roots and diazotrophs were analyzed in soils.

### 2.3. Soil Physicochemical Analysis

Soil pH was measured using a glass electrode with a soil-to-water ratio of 1:2.5. The soil organic matter (SOM) content was determined using the potassium dichromate oxidation method [28]. Total nitrogen (TN) and alkali hydrolyzable nitrogen (AN) were measured using the sulfuric acid extraction and alkaline diffusion methods, respectively. The total phosphorus (TP) and available phosphorus (AP) were determined using the molybdenum blue method [29]. Total potassium (TK) and available potassium (AK) were determined using flame photometry after extraction with sodium hydroxide and neutral ammonium acetate, respectively [30].

### 2.4. DNA Extraction and Amplicon Sequencing

Root samples were ground in liquid nitrogen. The DNA was extracted from the soil and roots using a Fast DNA^®^ SPIN Kit (MP Biomedicals, Santa Ana, CA, USA) and Plant DNA Extraction Mini Kit B (Mo Bio Laboratories, Inc., Carlsbad, CA, USA), respectively. The *nifH* amplicon for diazotrophs was amplified using the specific primer pair nifH-F (AAAGGYGGWATCGGYAARTCCACCAC) and nifH-R (TTGTTSGCSGCRTACATSGCCATCAT). The reaction volume for 20 μL included 0.8 μL of each 10 μM forward and reverse primer, 10 ng of template DNA, 4 μL of 5 × FastPfu Buffer, 2 μL of 2.5 mM dNTPs, 0.4 μL of FastPfu Polymerase, 0.2 μL of BSA, and added up to sterile water. The PCR condition was conducted at 95 °C for 3 min, then 40 cycles at 95 °C for 30 s, 60 °C for 30 s, 72 °C for 45 s, and final extension at 72 °C for 10 min.

The nested PCR was used to amplify the 18S rRNA gene fragments for root AMF community analysis. The primer set AML1 (ATCAACTTTCGATGGTAGGATAGA) and AML2 (GAACCCAAACACTTTGGTTTCC) were determined at the first PCR reaction and AMV4.5NF (AAGCTCGTAGTTGAATTTCG) and AMDGR (CCCAACTATCCCTATTAATCAT) was used to amplify at the second round PCR. The PCR reaction conditions and systems were described in our previous studies [24,26].

PCR products for *nifH* and 18S rRNA genes were pooled and purified. After that, sequencing was performed using the Illumina MiSeq PE300 platform (Illumina, San Diego, CA, USA) at Shanghai Majorbio Bio-Pharm Technology Co., Ltd. (Shanghai, China).

### 2.5. Sequence Analysis

The 18S rRNA of AMF and *nifH* gene sequences were processed in QIIME v2-2020.2, and the *NifH* Miseq Illumina Amplicon Analysis Pipeline (NifMAP) was used to remove sequences amplified from pseudogenes and homologs. The raw reads of both genes were imported into the QIIME environment. Amplicon sequence variants (ASVs) were denoised for raw sequences with the “q2-DADA2” plugin. The sequences of pseudogenes and homologs in *nifH* ASVs were then filtered against nucleotide-based HMM using the hmmsearch command in HMMER according to NifMAP. A naïve bayes classifier trained, with the PR2 and *nifH* reference database, was used to classify the ASVs of corresponding gene with the “q2-feature-classifier” plugin of QIIME2, respectively. All taxa were verified using the NCBI taxonomy database. Alpha diversity indices were calculated using normalized OTU tables in the R package “RAM” and further illustrated in the R package “ggplot2”. The ASV tables were further rarefied with the function “rarefy“ of the R package “vegan” and used to calculate alpha diversity indices with functions from the R package “RAM”.

### 2.6. Soil Physicochemical Analysis

#### 2.6.1. Plant Diversity Analysis

The plant richness (R) and Shannon index (H′) were calculated as follows:

R = the total number of plant species in each 10 m × 10 m plots

(1) $$H^{\prime }=-\sum_{i=1}^{S}Pi\;\ln\;Pi$$

where *S* is the total number of species in each plot, and *Pi* is the proportional density of individuals of the *i*^th^ species.

#### 2.6.2. Statistical Analysis

Significant differences in soil physiochemical properties, soil diazotrophs, root AMF, and plant diversity among slope positions were examined using one-way ANOVA and Duncan’s test at *p* < 0.05. Ternary plots were performed to identify the distribution of soil diazotroph and root AMF community composition across the upper, middle, and lower slopes. Random forest analysis was used to identify the main predictors of diazotroph abundance and root AMF diversity. The increase in mean square error (MSE) resulted in the need to quantify the relative importance of soil physiochemical property parameters and plant diversity using the ‘randomForest’ package [31]. Statistical analyses in the study were performed using R v4.02.

## 3. Results

### 3.1. Change in Soil Properties, Plant Diversity, and Soil Diazotroph and Root AMF Diversity

**Figure 1:**
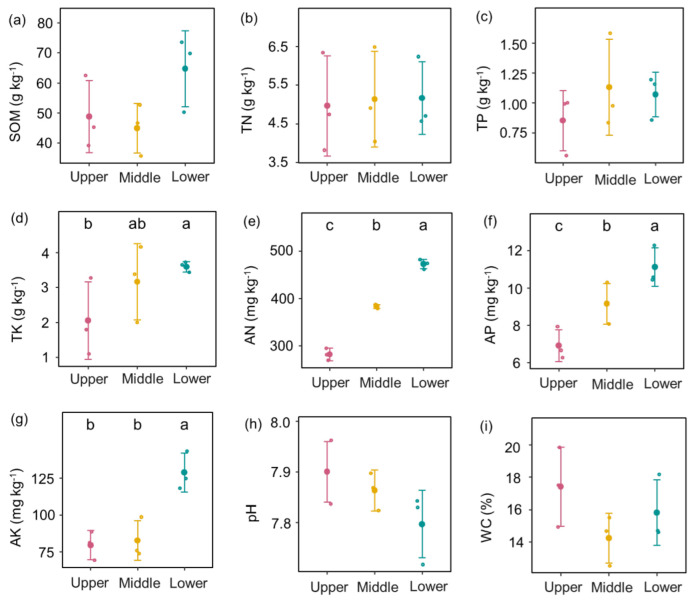
Differences in soil physiochemical properties among upper, middle, and lower slope positions. (**a**) Soil organic matter; (**b**) Total nitrogen; (**c**) Total phosphorus; (**d**) Total potassium; (**e**) Alkali hydrolyzable nitrogen; (**f**) Available phosphorus; (**g**) Available potassium; (**h**) soil pH; (**i**) water content. Values are means ± standard errors (*n* = 3). Different letters indicate statistical differences at *p* < 0.05 among upper, middle, and lower slope positions.

**Figure 2:**
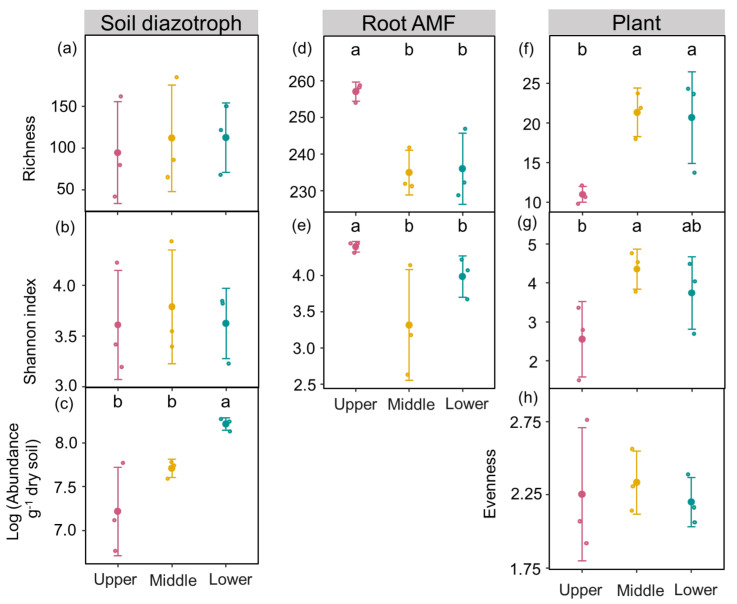
Soil diazotroph diversity and abundance, root AMF diversity, and plant diversity among upper, middle, and lower slope positions. (**a**) Diazotroph richness; (**b**) Diazotroph Shannon index; (**c**) Diazotroph abundance; (**d**) Root AMF richness; (**e**) Root AMF Shannon index; (**f**) Plant richness; (**g**) Plant Shannon index; (**h**) Plant evenness. Values are means ± standard errors (*n* = 3). Different letters indicate statistical differences at *p* < 0.05 among upper, middle, and lower slope positions.

The one-way ANOVAs of slope position showed that soil nutrients, soil diazotroph abundance, plant diversity, and root AMF diversity significantly differed among the lower, middle, and upper slopes. Specifically, TK, AN, AP, and AK were lower on the upper slope than on the lower slope (Figure 1). Soil pH and WC were similar among slope positions. Plant richness was lower on the upper slope than on the middle and lower slopes (Figure 1). Furthermore, the plant Shannon index was higher on the middle slope compared to the upper slope, while plant evenness was similar among slope positions (Figure 2).

Additionally, soil diazotroph abundance was higher on the lower slope compared to the upper and middle slopes, while no difference in diazotroph richness and the Shannon index was observed between the lower, middle, and upper slopes. Moreover, both root AMF richness and the Shannon index were higher on the upper slope than on the middle and lowerslopes (Figure 2).

### 3.2. Variations in Soil Diazotroph and Root AMF Community Compositions

**Figure 3:**
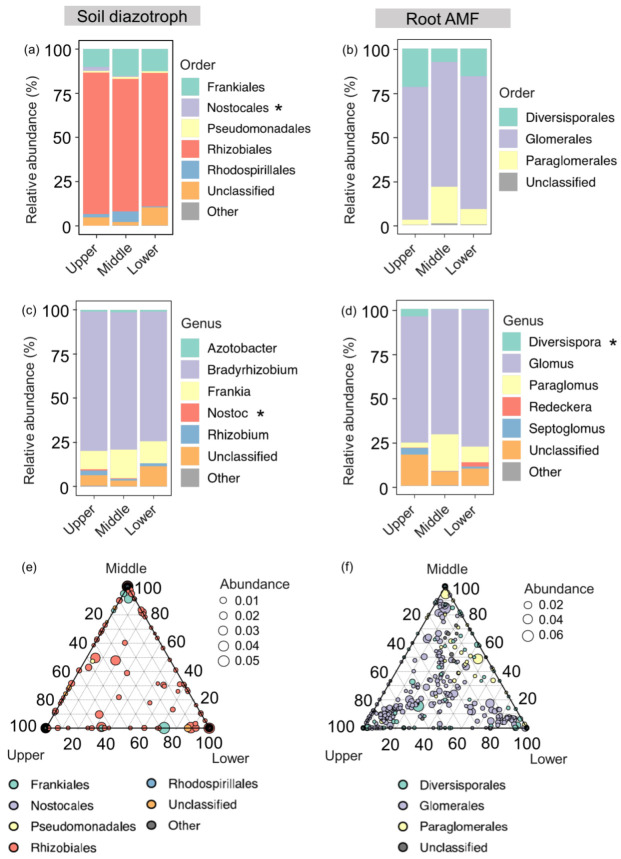
Soil diazotroph and root AMF community composition among slope positions. The relative abundance of the taxa at the levels of order (**a**,**b**) and genus (**c**,**d**). The asterisk “*” in the (**a**,**c**,**d**) denotes the significant difference among upper, middle, and lower slope positions. The ternary plots of all ASVs detected in soil diazotroph and root AMF (**e**,**f**); each circle represents one ASV, and the circle size indicates the relative abundance. The position of ASVs according to three axes were determined by the contribution of three slope positions to the total relative abundance and proximity to that vertex, indicating enrichment of that ASV in the slope position.

Diazotrophs were mainly identified for five taxa at the order level, with Frankiales, Nostocales, Pseudomonadales, Rhizobiales, and Rhodospirillales. Rhizobiales (76.7%), Frankiales (12.7%), and Rhodospirillales (2.8%) were the most abundant orders. Three AMF orders were identified from plant roots. Root AMF taxa were dominated by Glomerales (73.7%) and Diversisporales (15%) (Figure 3a,b). At the genus level, *Bradyrhizobium* (74.4%) and *Frankia* (12.7%) were the most abundant taxa for soil diazotrophs, while *Glomus* (72.5%) and *Paraglomus* (10.7%) were the dominant genera for root AMF (Figure 3c,d).

Soil diazotroph and root AMF community compositions showed significant variations among the three slope positions, suggesting that slope positions shifted the structure of soil diazotroph and root AMF species. Rhizobiales and Glomerales had the highest relative abundance on all three slope positions. The relative abundance of Nostocales and *Nostoc* were higher on the upper slope compared to the lower slope (Figure 3a,c). Ternary plots showed that Paraglomerales were enriched in the middle and lower slopes (Figure 3f).

### 3.3. Relationships between Soil Properties, Plant Diversity, and Soil Diazotroph and Root AMF Communities

**Figure 4:**
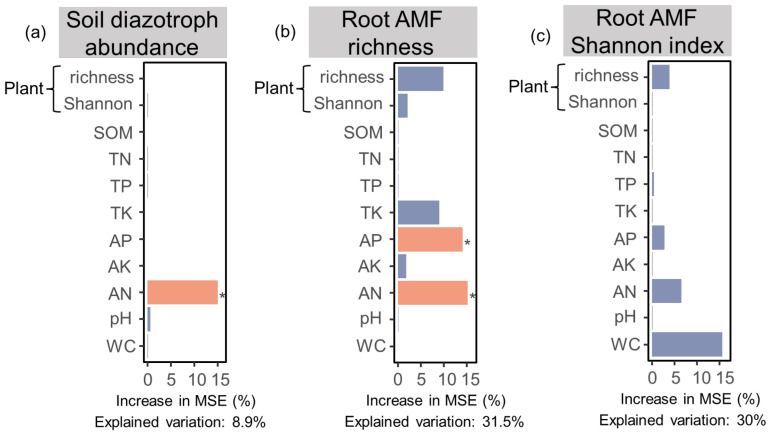
Relative importance of the soil physiochemical property parameters and plant diversities on the soil diazotroph abundance, root AMF richness, and Shannon index using random forests models. (**a**) Diazotroph abundance; (**b**) Root AMF richness; (**c**) Root AMF Shannon index. The asterisk “*” above the yellow indicates a significant effect.

The results from the random forest model analysis showed that 8.9%, 31.5%, and 30% of the variations in the soil diazotroph abundance, root AMF richness, and Shannon index were explained by soil properties and plant diversity, respectively. The AN was the main contributor to predicting soil diazotroph abundance. The plant richness, AP, and AN had a large contribution to variations in root AMF richness (Figure 4).

**Figure 5:**
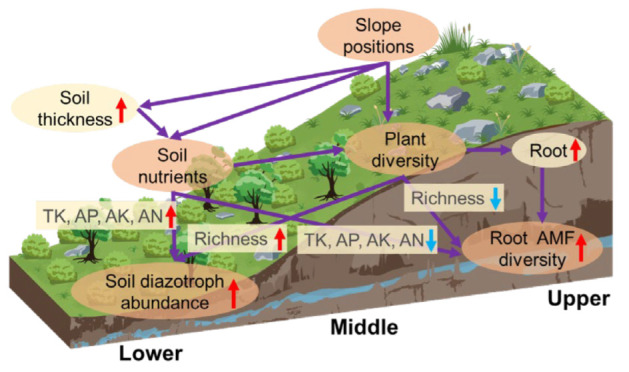
Conceptual model revealing the relationship between soil physiochemical property parameters and soil diazotroph as well as root AMF at different slope positions. TK: total potassium; AN: alkali-hydrolyzable nitrogen; AP: available phosphorus; AK: available potassium. The red arrows suggest an increase in effect, the blue arrows demonstrated declines, and the purple arrow shows how a physical condition or chemical condition impacts chemistry, AMF diversity, or diazotroph abundance.

Overall, high soil nutrient availability and plant richness increased soil diazotroph abundance. In contrast, low soil nutrient and plant diversity may induce root AMF diversity (Figure 5).

## 4. Discussion

### 4.1. Slope Position Effect on Soil Diazotroph and Root AMF Diversity

Topographic factors such as slope position have significant effects on the variability of subfactors (e.g., soil nutrients, plant diversity, and light), leading to variations in soil diazotrophs and root AMF communities [12,32]. Many studies have demonstrated that soils in lower foot slopes have great potential to induce C and N accumulation [33,34,35]. In this study, higher nutrient availability (e.g., TK, AN, AP, and AK) was likely in the lower position than in the upper slope (Figure 1) due to strong runoff and soil erosion, which enhanced residue and sediment removal from the upper slope to the lower slope [35,36,37]. Increased nutrient availability at lower slope positions was beneficial for plant growth [38], resulting in increased plant richness and the Shannon index for the lower or middle slopes compared to the upper slope (Figure 2). The lower slope showed higher plant richness than on the upper slope, but a similar level of plant evenness among slope positions, which suggested that the plant distributed evenly, and relatively equal numbers of species belong to each species in different slope positions. Therefore, rich plant diversity rather than plant evenness with high plant productivity may promote litter and root exudation inputs on lower slopes. Rich litter, nutrients, and root exudation that accumulate on slower slopes provide more energy for microbes, thereby improving diazotroph, the high population growth [16,17]. This was supported by the fact that diazotroph abundance on the lower slope was higher than that of the middle and upper slopes (Figure 2).

In contrast, considering the close relationship between plant communities and AMF diversity in plant roots, the opposite results were obtained here. Although plant richness was enriched on the lower slope, lower root AMF richness and the Shannon index were observed on the middle and lower slopes compared with the upper slope (Figure 2). The slope position influences the root AMF diversity in several ways. First, karst ecosystems on the upper slopes have high gravel content and bedrock outcrops; therefore, the water storage capacity of the upper slope position is poor, thereby limiting vegetation recovery [22,39]. Thus, the plant community on the upper slope remained in the succession stage of grass; however, the shrubs performed well on the lower slope. The level of light reaching both the photoreceptors on the plants and the soil was more heterogeneous on the lower slope than on the upper slope because of vegetation heterogeneity on the lower slope. Under such conditions, there may be less light on the lower slope due to shading caused by the presence of taller and more abundant plants. In contrast, sufficient light can be provided on the upper slope because low plant cover helps to receive sunlight [40]. A study found that upper slopes with more light stimulated AMF diversity [12], which is consistent with our results. Second, owing to the shallow soil layer, plant roots were mainly distributed on the surface layer. High plant root densities acted as sources for some AMF species in plant root on the upper slope despite low plant diversity. This may explain the rich root AMF diversity on the upper slopes [41]. Moreover, a lower AP on the upper slopes compared with the lower slopes would consequently induce more root AMF species to absorb and transport AP, and to accelerate the plant growth tolerance under poor soil nutrient environments [1,4]. This finding suggests that harsh environmental filtering with low soil nutrients and plant diversity on the upper slope may increase the dependence of root AMF species to establish suitable habitats.

### 4.2. Responses of Soil Diazotroph and Root AMF Community Compositions to Slope Position

Understanding the microbial compositions at different slope positions is essential to predict the adaptive strategies of soil diazotrophs and root AMF groups on the upper, middle, and lower slopes. Taxonomic profiling revealed that Rhizobiales and Glomerales were the most abundant orders, accounting for 77% and 74% of the total sequences for soil diazotroph and root AMF, respectively (Figure 3). This was similar to our previous studies on karst grassland soil [24,42]. Both Rhizobiales and Glomerales are well-known orders that can colonize plant roots and have a strong ability to adapt to ecological conditions in severe environments, such as karst ecosystems with high pH and Ca [23,43,44]. In addition, they can alleviate nutrient limitations by enhancing nutrient exchange [26]. Collectively, these dominant taxa could improve their ecological functions to regulate nutrient cycling in fragile karst ecosystems.

The slope position showed potential impacts on soil diazotroph and root AMF community compositions. Specifically, the observed species distribution was different within the upper, middle, and lower slopes. The relative abundances of the order Nostocales and genus *Nostoc* were higher in the upper slopes than the lower slopes. This could be explained by the fact that *Nostoc* that belongs to Nostocales performs well in extreme situations [45,46], such as upper slopes with poor water-holding environments because of the shallow and discontinuous soil. Moreover, Nostocales also participate in the formation of biological crusts [47]. Generally, biological crusts occur frequently in grasslands due to suitable shading and sunlight, leading to a high abundance of Nostocales on the upper slope [46]. For root AMF species, Paraglomerales play an important role in plant nutrient absorption and transfer under low AP levels, especially in the soil rhizosphere [48,49,50]. This study confirmed that Paraglomerales were rich in the middle and upper slopes, with low AP, with more abundant plant roots than that of the lower slopes (Figure 3). Interestingly, the dominant orders of Rhizobiales and Glomerales were rich in the upper, middle, and lower slopes. This result suggests that high proportions of Rhizobiales and Glomerales can survive over a wide range of nutrient gradients.

### 4.3. Slope Position with Different Soil Nutrients and Plant Diversity Driving Soil Diazotroph and Root AMF Properties

A random forest model was constructed to better understand the main contribution of soil properties and nutrients. Many previous studies reported that soil environment conditions (e.g., moisture) and nutrients (e.g., AP and AN) were strongly related to diazotroph abundance and diversity [2,25,46]. In the present study, the variation in diazotroph abundance was explained more by AN (Figure 4). The early stage of vegetation succession in the karst region was still N limited, owing to tillage disturbances which accelerated the subsurface leaching of N before vegetation restoration [51]. Increased N availability on the lower slopes could improve plant growth with high plant productivity and diversity. This increases root exudates which provide C resources for diazotrophs, and in turn stimulates diazotroph growth (Figure 5) [33,52,53]. These results suggest that increasing soil nutrients on the lower slope, especially with high AN, could indirectly improve diazotroph abundance by promoting plant growth.

Plant richness, AP, and AN were the main contributors to root AMF richness and the Shannon index variations (Figure 4). The upper slope was characterized by low plant richness, AP, and AN, which could increase their richness to increase nutrient supply [4,54].

In addition, as mentioned above, richer plant root biomass in the surface soil due to the shallow soil layer leads to poorer water-holding capacity on the upper slope than on the lower slope. Additional AMF species in the plant roots were selected by enhancing cooperative relationships among root AMF taxa when the plant root biomass was increased in the surface soil on the upper slopes; this emphasized the important effect of plant root biomass on root AMF diversity. Consequently, the deterministic assembly factors for the variations in soil diazotroph abundance and root AMF diversity can be partially explained by environmental heterogeneity along the slope gradients (Figure 5).

## 5. Conclusions

This study expands our understanding of soil diazotrophs and root AMF communities in response to slope position during vegetation recovery. Soil nutrients and plant properties were the main factors driving the variation in community composition, and the diversity of diazotrophs and AMF. High N availability stimulates plant growth, thereby increasing diazotroph abundance, owing to sufficient litter and root exudate inputs. High light and low nutrient levels could promote AMF diversity in plant roots on the upper slope. Moreover, the slope position indirectly affected the diazotroph and AMF community composition. For example, the relative abundances of the order level for the diazotroph Nostocales and AMF Paraglomerales on the upper slope were richer than the lower slope. Overall, our results emphasize that slope position indirectly influences soil diazotroph abundance and root AMF diversity by regulating soil nutrients, microclimate conditions, and plant roots. However, future studies should evaluate their important roles in organic matter decomposition and N fixation along the slope gradients.

## Data Availability

The data is not public, and can be requested from the author if necessary.
